# Supplementary figures and images for: Antibiotics affect the pharmacokinetics of n-butylphthalide in vivo by altering the intestinal microbiota
Source: PLoS One. 2024 Jun 25;19(6):e0297713. doi: 10.1371/journal.pone.0297713 (PMC11198832; doi:10.1371/journal.pone.0297713)

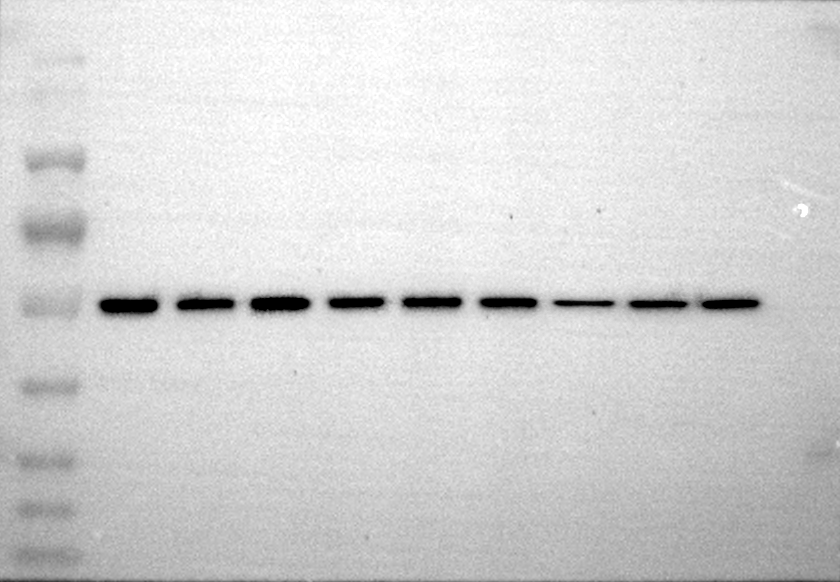

Supplement: S1 File — (ZIP) [file pone.0297713.s002.zip › WB original images/CYP3A1-1.tif]

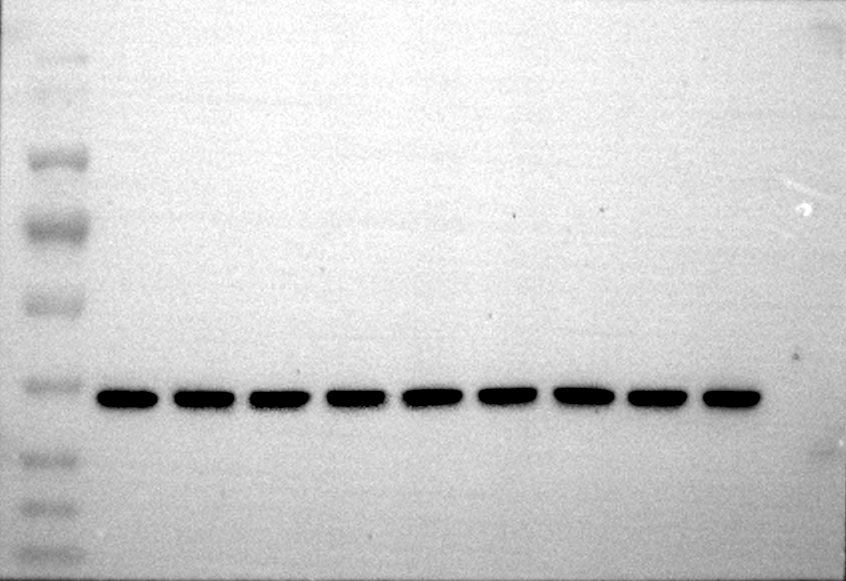

Supplement: S1 File — (ZIP) [file pone.0297713.s002.zip › WB original images/CYP3A1-1-GAPDH.tif]

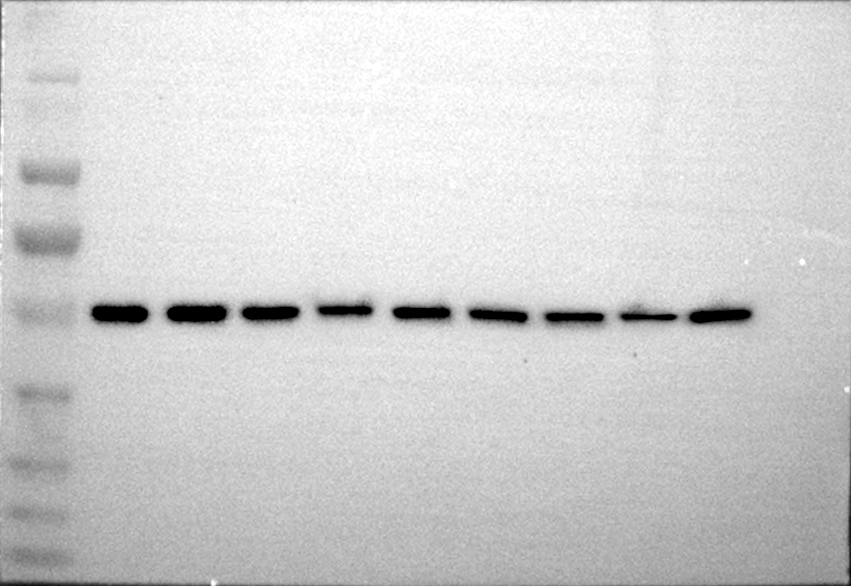

Supplement: S1 File — (ZIP) [file pone.0297713.s002.zip › WB original images/CYP3A1-2.tif]

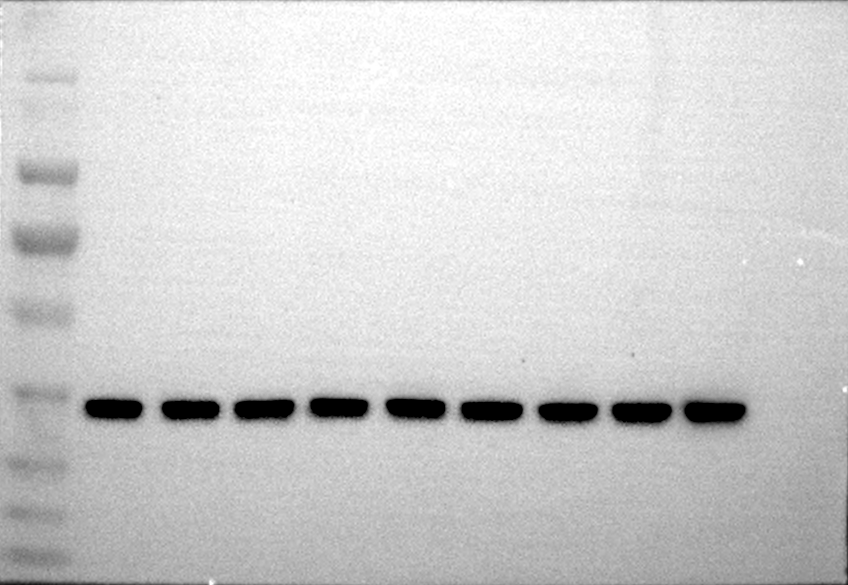

Supplement: S1 File — (ZIP) [file pone.0297713.s002.zip › WB original images/CYP3A1-2-GAPDH.tif]
